# Detection of the Diversity of Cytoplasmic Male Sterility Sources in Broccoli (*Brassica Oleracea* var. *Italica*) Using Mitochondrial Markers

**DOI:** 10.3389/fpls.2016.00927

**Published:** 2016-06-24

**Authors:** Jinshuai Shu, Yumei Liu, Zhansheng Li, Lili Zhang, Zhiyuan Fang, Limei Yang, Mu Zhuang, Yangyong Zhang, Honghao Lv

**Affiliations:** Institute of Vegetables and Flowers, Chinese Academy of Agricultural SciencesBeijing, China

**Keywords:** broccoli, cytoplasmic male sterility, origin, mitochondrial markers, diversity

## Abstract

Broccoli (*Brassica oleracea* var. *italica*) is an important commercial vegetable crop. As part of an efficient pollination system, cytoplasmic male sterility (CMS) has been widely used for broccoli hybrid production. Identifying the original sources of CMS in broccoli accessions has become an important part of broccoli breeding. In this study, the diversity of the CMS sources of 39 broccoli accessions, including 19 CMS lines and 20 hybrids, were analyzed using mitochondrial markers. All CMS accessions contained the *ogu orf138*-related DNA fragment and the key genes of *nap* CMS, *pol* CMS, and *tour* CMS were not detected. The 39 CMS accessions were divided into five groups using six *orf138*-related and two simple sequence repeat markers. We observed that *ogu* CMS R_3_ constituted 79.49% of the CMS sources. CMS6 and CMS26 were differentiated from the other accessions using a specific primer. CMS32 was distinguished from the other accessions based on a 78-nucleotide deletion at the same locus as the *orf138*-related sequence. When the coefficient was about 0.90, five CMS accessions (13CMS6, 13CMS23, 13CMS24, 13CMS37, and 13CMS39) exhibiting abnormal floral organs with poor seed setting were grouped together. The polymerase chain reaction amplification profiles for these five accessions differed from those of the other accessions. We identified eight useful molecular markers that can be used to detect CMS types during broccoli breeding. Our data also provide important information relevant to future studies on the possible origins and molecular mechanisms of CMS in broccoli.

## Introduction

Broccoli (*Brassica oleracea* var. *italica*) is an economically important vegetable crop that displays heterosis (Mao et al., 2012; Walley et al., 2012). The use of cytoplasmic male sterility (CMS) to produce hybrid seed can prevent self-pollination of female parents and lead to hybridization rates of up to 100%. The application of CMS provides an alternative to using self-incompatible lines during crop breeding (Wang et al., 2012). Currently, CMS is widely used for hybrid breeding and seed production of important vegetable crops, including onion (Havey, 2000), cabbage (Fang et al., 2001, 2004), radish (Lee et al., 2008), and cauliflower (Dey et al., 2011; Kaminski et al., 2012; Bhatia et al., 2015). Similarly, CMS lines play a crucial role in broccoli breeding and hybrid production (Mao et al., 2012; Shu et al., 2014; Jing et al., 2015). However, the morphologies of CMS floral organs usually exhibit complex variations according to genetic backgrounds and CMS types during the transfer process (Zhu et al., 1998), and the variations could considerably affect the efficiency of pollination and seed production (Shu J. S. et al., 2015). *Brassica* species are a rich source of different types of CMS, including *ogu* CMS (Ogura, 1968), *pol* CMS (Fu et al., 1995), *nap* CMS (Thompson, 1972), *nig* CMS (Pearson, 1981), and *hau* CMS (Wan et al., 2008), which are all widely used. Domestic and international breeding organizations currently cross CMS materials with each other to transfer CMS to broccoli. However, a lack of information regarding the types of CMS has made the development of broccoli lines with ideal CMS relatively difficult. The rapid identification of CMS sources and types would provide valuable information relevant to the introduction of CMS into broccoli and genetic improvement of this important crucifer.

Expression of specific mitochondrial genes is highly correlated with CMS, exactly which mitochondrial genes are important depends on species in which CMS source originated (Schnable and Wise, 1998; Hanson and Bentolila, 2004). However, most of the genes exhibit a maternal inheritance pattern in *Brassica* crops (Reboud and Zeyl, 1994). *orf138, orf224-atp6, orf263, orf222*, and *orf288* are key genes of *ogu* (Bonhomme et al., 1992), *pol* (Singh and Brown, 1993), *tour* (Landgren et al., 1996), *nap* (L'Homme et al., 1997), and *hau* (Heng et al., 2014) CMS, respectively. Therefore, specific primers designed based on these mitochondrial genomic regions can be used to distinguish CMS types. Complete mitochondrial genomes of *Arabidopsis thaliana, B. napus* (*nap* and *pol*), *B. rapa* (*cam*), *B. oleracea, B. juncea*, and *B. carinata* have been determined (Unseld et al., 1997; Handa, 2003; Chang et al., 2011; Chen et al., 2011; Heng et al., 2014), with the increasing number of mitochondrial nucleotide sequences, many mitochondrial markers have been developed to identify CMS types in *B. juncea* (Ashutosh et al., 2005; Wan et al., 2008; Yu et al., 2014), *B. napus* (Wei et al., 2005; Wan et al., 2008), *B. oleracea* (Li et al., 2009; Zhang et al., 2010, 2011; Wang et al., 2012), *B. oleracea* var. *italica* (Jing et al., 2015), and *B. rapa* (Zhang et al., 2007; Shi et al., 2012; Heng et al., 2015). The markers have also been used to analyze genetic diversity in varieties of *B. napus* (Handa, 2007; Zhao et al., 2010) and *B. rapa* (Zhang et al., 2013), and to differentiate between somatic hybrids of *B. juncea* and *B. oleracea* (Lian et al., 2011). Thus, far, mitochondrial markers have not been successfully used to analyze the different types of CMS and genetic diversity of original sources of CMS in broccoli.

We completed this study to address the issues involved in selecting suitable CMS sources for broccoli breeding. Our objectives were as follows: (1) distinguish the cytoplasm types of 19 CMS lines and 20 hybrids using mitochondrial molecular markers, (2) reveal the diversity in the cytoplasm types of CMS in broccoli, and (3) confirm the polymorphic loci by cloning and sequencing.

## Materials and methods

### Plant materials and DNA extraction

Details of the plant materials used in this study are provided in Table 1. We analyzed 40 broccoli CMS accessions (13CMS1–13CMS39, 13ML), including 19 CMS lines (13CMS1–13CMS19) and 20 hybrids (13CMS20–13CMS39) with different origins, and one inbred line (13ML) as a reference. The original CMS sources and hybrids were obtained from research institutes and seed companies from all over the world. 14CMS1-39 and 15CMS1-39 were obtained from 13CMS1-39 and 14CMS1-39 through backcrossing, respectively. The recurrent male parent was broccoli inbred line B59 (Supplementary Table 1). All accessions were grown in experimental greenhouses of the Institute of Vegetables and Flowers, Chinese Academy of Agricultural Sciences, Beijing, China in 2013–2015. For each accession, total genomic DNA was isolated from eight to ten true leaves using a modified hexadecyltrimethylammonium bromide method (Murray and Thompson, 1980) and stored at −30°C.

**Table 1:** **Broccoli CMS accessions and origins used in this study**.

**Field code**	**Line name**	**Type**	**Backcross generations**	**Origin of cytoplasmic male sterile sources**
13CMS1	OguraCMS R3-B59^a^	Cytoplasmic male sterile line	BC16	Variety introduction (Asgrow seed Co., US)
13CMS2	CMS04-B59	Cytoplasmic male sterile line	BC9	Variety introduction (Shanghai Horticultural Research Institute, China)
13CMS3	CMS07-B59	Cytoplasmic male sterile line	BC9	Variety introduction (Beijing Vegetable Research Center, China)
13CMS4	CMS12-B59	Cytoplasmic male sterile line	BC7	Variety introduction (Shanghai Horticultural Research Institute, China)
13CMS5	CMS13-B59	Cytoplasmic male sterile line	BC8	Variety introduction (Beijing Vegetable Research Center, China)
13CMS6	CMS132-B59	Cytoplasmic male sterile line	BC8	Variety introduction (Japan)
13CMS7	CMS724-B59	Cytoplasmic male sterile line	BC7	Variety introduction (Taiwan, China)
13CMS8	CMS727-B59	Cytoplasmic male sterile line	BC8	Variety introduction (Seminis Seeds Co., Ltd., USA)
13CMS9	CMS736-B59	Cytoplasmic male sterile line	BC7	Variety introduction (Japan)
13CMS10	CMS738-B59	Cytoplasmic male sterile line	BC7	Variety introduction (Japan)
13CMS11	CMSGD-B59	Cytoplasmic male sterile line	BC6	Variety introduction (Guangdong, China)
13CMS12	CMS1190-B59	Cytoplasmic male sterile line	BC4	Variety introduction (Beijing Honor Seeds Co., Ltd., China)
13CMS13	CMS1162-B59	Cytoplasmic male sterile line	BC4	Variety introduction (Kunming Kunhua Seed Co., Ltd., China)
13CMS14	CMS1166-B59	Cytoplasmic male sterile line	BC4	Variety introduction (Kunming Kunhua Seed Co., Ltd., China)
13CMS15	CMS1169-B59	Cytoplasmic male sterile line	BC4	Variety introduction (Kunming Kunhua Seed Co., Ltd., China)
13CMS16	CMS1176-B59	Cytoplasmic male sterile line	BC4	Variety introduction (Kunming Kunhua Seed Co., Ltd., China)
13CMS17	CMS1177-B59	Cytoplasmic male sterile line	BC4	Variety introduction (Kunming Kunhua Seed Co., Ltd., China)
13CMS18	CMS1183-B59	Cytoplasmic male sterile line	BC4	Variety introduction (Beijing, China)
13CMS19	CMSYB6-B59	Cytoplasmic male sterile line	BC2	Variety introduction (Japan)
13CMS20	CMSLvXinErHao	Hybrid	−	Variety introduction (Taiwan Suntech Seed Co., Ltd., China)
13CMS21	CMSYouSheng	Hybrid	−	Variety introduction (Wong Ching Ho Co., Ltd., Hong Kong, China)
13CMS22	CMSYuXi	Hybrid	−	Variety introduction (Kunming Kunhua Seed Co., Ltd., China)
13CMS23	CMSWeiJingLv	Hybrid	−	Variety introduction (Tianhe agricultural companies, Hong Kong, China)
13CMS24	CMSWangLv	Hybrid	−	Variety introduction (Japan)
13CMS25	CMSB2944	Hybrid	−	Variety introduction (Wei Qin Enterprises Ltd., Hong Kong, China)
13CMS26	CMSMarathon	Hybrid	−	Variety introduction (Japan)
13CMS27	CMSB2946	Hybrid	−	Variety introduction (Beijing Honor Seeds Co., Ltd., Beijing, China)
13CMS28	CMSB2947	Hybrid	−	Variety introduction (Beijing Honor Seeds Co., Ltd., Beijing, China)
13CMS29	CMSLD66	Hybrid	−	Variety introduction (Korea)
13CMS30	CMSB2949	Hybrid	−	Variety introduction (Tianjin Kernel Vegetable Research Institute, China)
13CMS31	CMSB2950	Hybrid	−	Variety introduction (Tianjin Kernel Vegetable Research Institute, China)
13CMS32	CMSXiLanHuaErHao	Hybrid	−	Variety introduction (Seminis Seeds Co., Ltd., USA)
13CMS33	CMSB2952	Hybrid	−	Variety introduction (Wong Ching Ho Co., Ltd., Hong Kong, China)
13CMS34	CMS YouXiu	Hybrid	−	Variety introduction (Japan)
13CMS35	CMSB2069	Hybrid	−	Variety introduction (Sakata Seed Corporation, Japan)
13CMS36	CMSB2071	Hybrid	−	Variety introduction (Seminis Seeds Beijing Co., Ltd., Beijing, China)
13CMS37	CMSB2072	Hybrid	−	Variety introduction (Taiwan Ho-Huan Agricultural Product Co., Ltd., China)
13CMS38	CMSB2074	Hybrid	−	Variety introduction (Syngenta China Company, Beijing, China)
13CMS39	CMSB2075	Hybrid	−	Variety introduction (Seminis Seeds Beijing Co., Ltd., Beijing, China)
13ML	B59	Inbred	−	Institute of Vegetables and Flowers, Chinese Academy of Agricultural Sciences (Beijing, China)

a Number refers to the genotype of the backcross parents; –, no backcross generations.

### Polymerase chain reaction and sequence analysis

We used the following primers (Table 2): one pair of primers specific for *orf138* (Zhang et al., 2013) and six pairs of primers designed based on *orf* 138-related genomic sequences (GenBank GI: 288879, *Raphanus sativus* mitochondrial *orf138, orfB*, and *trnfM*; P8–P13); one pair of primers specific for *orf222*–*orf224* (Zhang et al., 2010), *orf224* (Wei et al., 2005), *atp6-orf 224* (Shi et al., 2012), or *orf263* (Shi et al., 2012); two pairs of primers specific for *orf222* (Wei et al., 2005; Li et al., 2009); and six pairs of mitochondrial simple sequence repeat (SSR) primers (Honma et al., 2011; Wang et al., 2012). All primers were synthesized by Bo Maide Biotech Co., Ltd. (Beijing, China).

**Table 2:** **Sequences and details of the primers used in this study**.

**Name**	**Sequence 5′-3**	**Annealing temperature**	**Targets/Locations**	**References**
P1F	GAAACGGGAAGTGACAAT	54°C	*orf138*	Zhang et al., 2013
P1R	GCATTATTTTCTCGGTCCAT			
P2F	AGCTGTCTGGAGGGAATC	54°C	*orf222*	Wei et al., 2005
P2R	GCGGTCTCACGCACTAATC			
P3F	ATGCCTCAACTGGATAAAT	55°C	*orf222*	Li et al., 2009
P3R	TCATCGAAATAGATCGAGTA			
P4F	GCCTCAACTGGATAAATTC	54°C	*orf222-orf224*	Zhang et al., 2010
P4R	CAAGGATCTCGTTCACCT			
P5F	AGCTGTCTGGAGGGAATC	55°C	*orf224*	Wei et al., 2005
P5R	ACGACATCAAGGAGGAAC			
P6F	TGAAATGGGAGGTCAGAAGC	56°C	*atp6-orf 224*	Shi et al., 2012
P6R	AAAAGGTGCTAACGGCAGTG			
P7F	ATGAAAAATAGACTCCAA	56°C	*orf263*	Shi et al., 2012
P7R	TCAGTCTAGATAATGCCG			
P8F	GCAATGATTACCTTTTTCGA	55°C	*orf138*	Li et al., 2009
P8R	GCATTATTTTCTCGGTCCAT			
P9F	GAAACGGGAAGTGACAATA	55°C	*orf138*	Wei et al., 2005
P9R	GCATTATTTTCTCGGTCCAT			
P10F	CCATATTTGGCTAAGCTGGTTTTCT	57°C	*orf138*	Zhang et al., 2010
P10R	TATTTTCTCGGTCCATTTTCCAC			
P11F	GCCCATATTTGGCTAAGCTG	56°C	*orf138*	Shi et al., 2012
P11R	TTTTCTCGGTCCATTTTCCA			
P12F	CGGTCGGTGTCCAAGATTT	58°C	*orf138*	
P12R	ACTGTTGGGGTCCTTGCTCT			
P13F	AATGAAGCTGTCTGGAGGGA	56°C	*orf138*	
P13R	TTCATTGAATACTTCCATACCTG			
P14F	CCGTTAGGGGTATTTAGTAACTCG	56°C	BnTR1	Honma et al., 2011
P14R	ACATAATGGCAATGTATCGGACTG			
P15F	GAAGTCCGAGGACCTTTAGTACC	56°C	BnTR4	
P15R	AGTAAGTTGTAGGTAGGGGCTTCAT			
P16F	ACCAAGATTGAGCCAGAT	55°C	*orf125*	Wang et al., 2012
P16R	CGTCCACTACCGAAAGAG			
P17F	CCCGAGAAGCACTGTTGA	55°C	*trnY-trnD*	
P17R	ACGGAGTGACAAAGGAGC			
P18F	CCTTCTGGGTTGACTTGA	55°C	*nad4L-orf101b*	
P18R	AGTGGTGCCCTCCTCTAA			
P19F	GCTGCTCATCACTACCTG	55°C	*orf448-nad6*	
P19R	CACTACGCTCACTGAAACTA			

Polymerase chain reaction (PCR) amplifications were performed in a final reaction volume of 20 μl, which consisted of 10 μl Dream Taq™ Green PCR Master Mix (Thermo Fisher Scientific Inc., Waltham, MA, USA), 1 μl primer (10 pmol/μl), and 2 μl (50 ng/μl) genomic DNA. The PCR program was as follows: 95°C for 5 min; 35 cycles of 95°C for 30 s, a suitable annealing temperature for 30 s, 72°C for 30–90 s; and a final 72°C for 10 min. The PCR was completed using an ABI Veriti 96-well PCR thermal cycler (Applied Biosystems, Foster City, CA, USA). The amplification products were analyzed on 3.0% (w/v) agarose gels in 0.5 × TBE buffer and visualized using the GoldView staining solution (Solarbio Technology Co., Ltd., Beijing, China). Stained gels were viewed and photographed under ultraviolet light using the Universal Hood II (Bio-Rad, Hercules, CA, USA).

Each polymorphic marker was tried to repeat amplification for three times, and for each polymorphic locus, the cloned products were separated and sequenced by Bo Maide Biotech Co., Ltd. Sequence alignment analyses were completed using the alignment tools of the Universal Protein Resource (http://www.uniprot.org/align/).

### Cluster analyses

We constructed a binary matrix by converting the polymorphic loci data into “1” or “0,” each DNA band generated was visually scored as an independent locus (1 for presence and 0 for absence). Qualitative differences in band intensities were not considered. Then completed the Unweighted Pair Group Method with Arithmetic Mean cluster analyses using NTSYSpc, version 2.11 software (Applied Biostatistics Inc., Exeter Software, Setauket, New York, USA). The genetic distances were calculated with the SM coefficient.

## Results

### Origins of CMS in broccoli accessions determined using mitochondrial molecular markers

**Figure 1:**
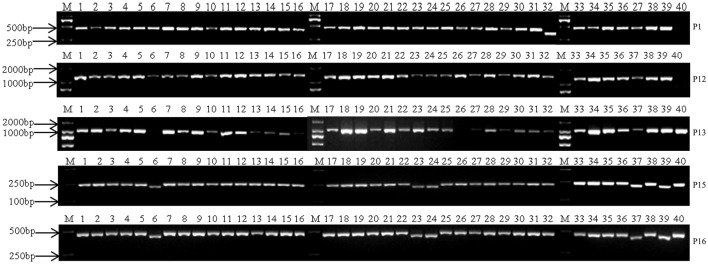
**PCR amplification profiles for 40 broccoli accessions using primer P1, P12, P13, P15, P16**. M, marker; 1–39, 13CMS1–13CMS39; 40, 13ML.

We screened 19 CMS lines (13CMS1–13CMS19; Table 1) and 20 CMS hybrids (13CMS20–13CMS39; Table 1) with thirteen pairs of mitochondrial DNA-specific primers (P1–P13; Table 2). Primer P1 exhibited polymorphism between CMS accessions and the inbred line (Figure 1). The fragment amplified by primer P1 was about 390 bp in 13CMS32 and 460 bp in 13CMS1–13CMS31 and 13CMS33–13CMS39. The results for primers P8–P11 were similar to those of primer P1. Primers P8–P11 did not generate an amplification product for 13ML, but did for the 39 CMS accessions. Additionally, the amplified product for 13CMS32 differed from those of the other 38 CMS accessions. Primer P12 amplified a 1200 bp product for 13CMS1–39, but did not amplify anything for 13ML (Figure 1). Primer P13 did not amplify any gene fragment for 13CMS6 and 13CMS26. However, an ~1000 bp fragment was amplified for the other accessions (Figure 1). Taken together, these results indicated the CMS of the 39 accessions was derived from *ogu* (*Ogura*) CMS, but there were differences in the nucleotide sequences among the accessions.

### Origins of CMS in broccoli accessions determined using mitochondrial SSR molecular markers

We used six pairs of mitochondrial SSR primers (P14–P19, Table 2) to amplify genomic DNA from the 40 broccoli accessions (13CMS1–13CMS39, 13ML; Table 1). The primers were developed based on the *B. napus* mitochondrial genome and effectively amplified *Brassica* fragments (Honma et al., 2011; Wang et al., 2012). All primers successfully amplified a gene fragment. However, only primers P15 and P16 exhibited clear polymorphisms (Figure 1). The amplicons produced by P15 for 13CMS6, 13CMS23, 13CMS24, 13CMS37, and 13CMS39 were about 220 bp, while those of the other broccoli accessions and 13ML were about 250 bp (Figure 1). The amplicons generated by P16 for 13CMS6, 13CMS23, 13CMS24, 13CMS37, and 13CMS39 were about 420 bp, while those of the other broccoli accessions and 13ML were ~470 bp (Figure 1). Our results indicated that the original CMS source for 13CMS6, 13CMS23, 13CMS24, 13CMS37, and 13CMS39 was the same, but differed from that of the other 34 CMS accessions.

### Validation of the polymorphic markers

In 2014 and 2015, we used nine polymorphic primers (P1, P8–P13, P15, and P16; Table 2) to screen another 80 broccoli accessions (14CMS1–14CMS39, 14ML, 15CMS1–15CMS39, 15ML; Supplementary Table 1) to confirm the accuracy of the polymorphic markers. The results we obtained were the same as those from 2013, which demonstrated the stability of the molecular markers developed in this study.

### Diversity of CMS origins in broccoli accessions

**Figure 2:**
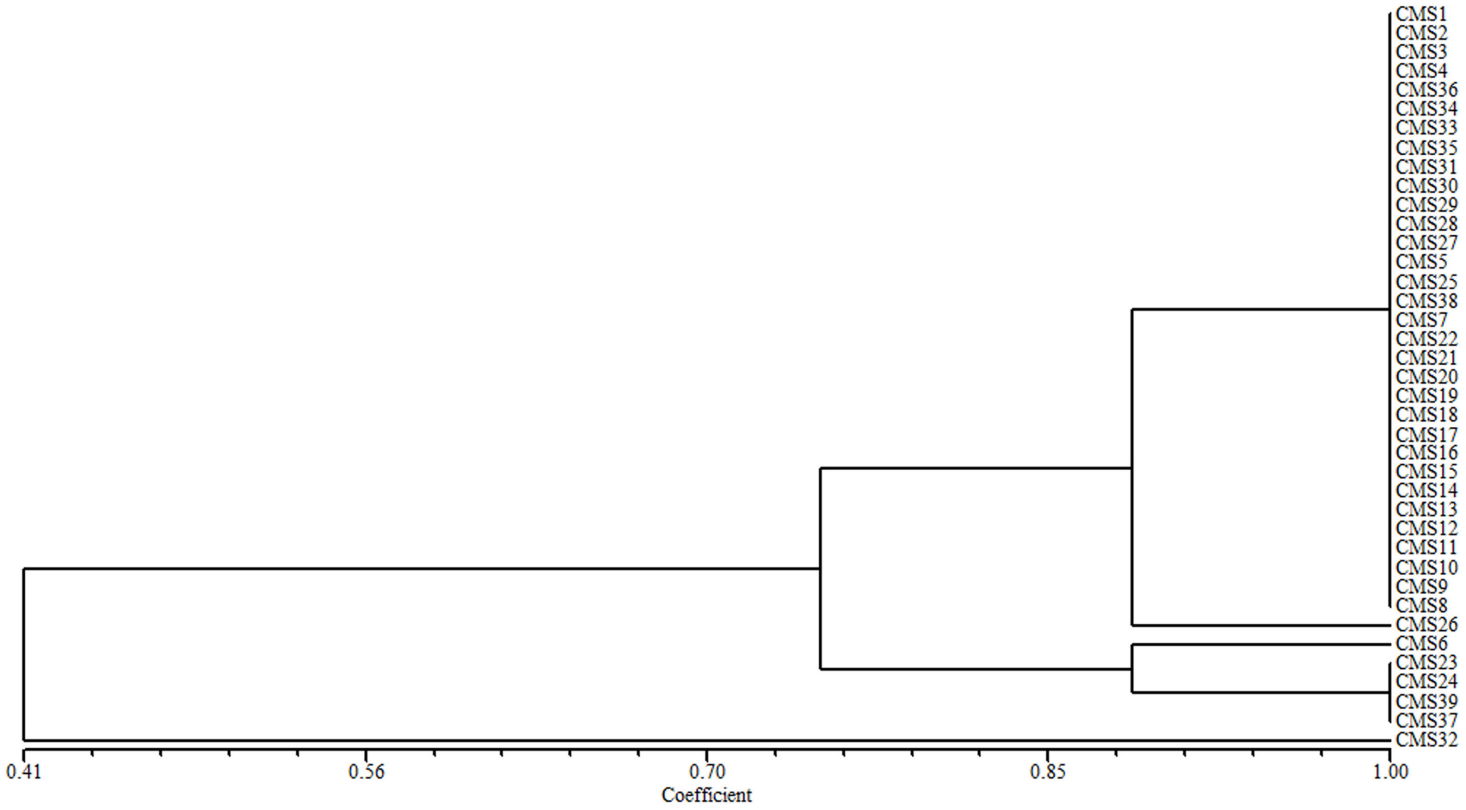
**Cluster analysis of the CMS origins for 39 broccoli accessions based on *ogu* CMS mitochondrial DNA-specific markers and ***B***. ***napus*** mitochondrial SSR markers**.

The 39 broccoli CMS accessions could be categorized into separate groups using *ogu* CMS mitochondrial DNA-specific markers or *B. napus* mitochondrial SSR markers. Combining the two types of analytical methods may be necessary to evaluate the diversity of CMS origins in broccoli accessions. A cluster analysis using all of the *ogu* CMS mitochondrial DNA-specific and *B. napus* mitochondrial SSR polymorphic markers revealed that the 39 CMS broccoli accessions could be divided into five groups (coefficient > 0.89) based on the origins of their CMS. We determined that CMS6, CMS26, and CMS32 each formed its own group, CMS23, CMS24, CMS37, and CMS39 belonged to one group, and the other 32 CMS accessions formed another group (coefficient > 0.89). When the coefficient was larger than 0.745, the 39 CMS accessions could be divided into three groups, CMS32 formed its own group, CMS6, CMS23, CMS24, CMS37, and CMS39 belonged to one group, and the other 33 CMS accessions formed another group (Figure 2).

### Sequence features of the polymorphic amplicons

**Figure 3:**
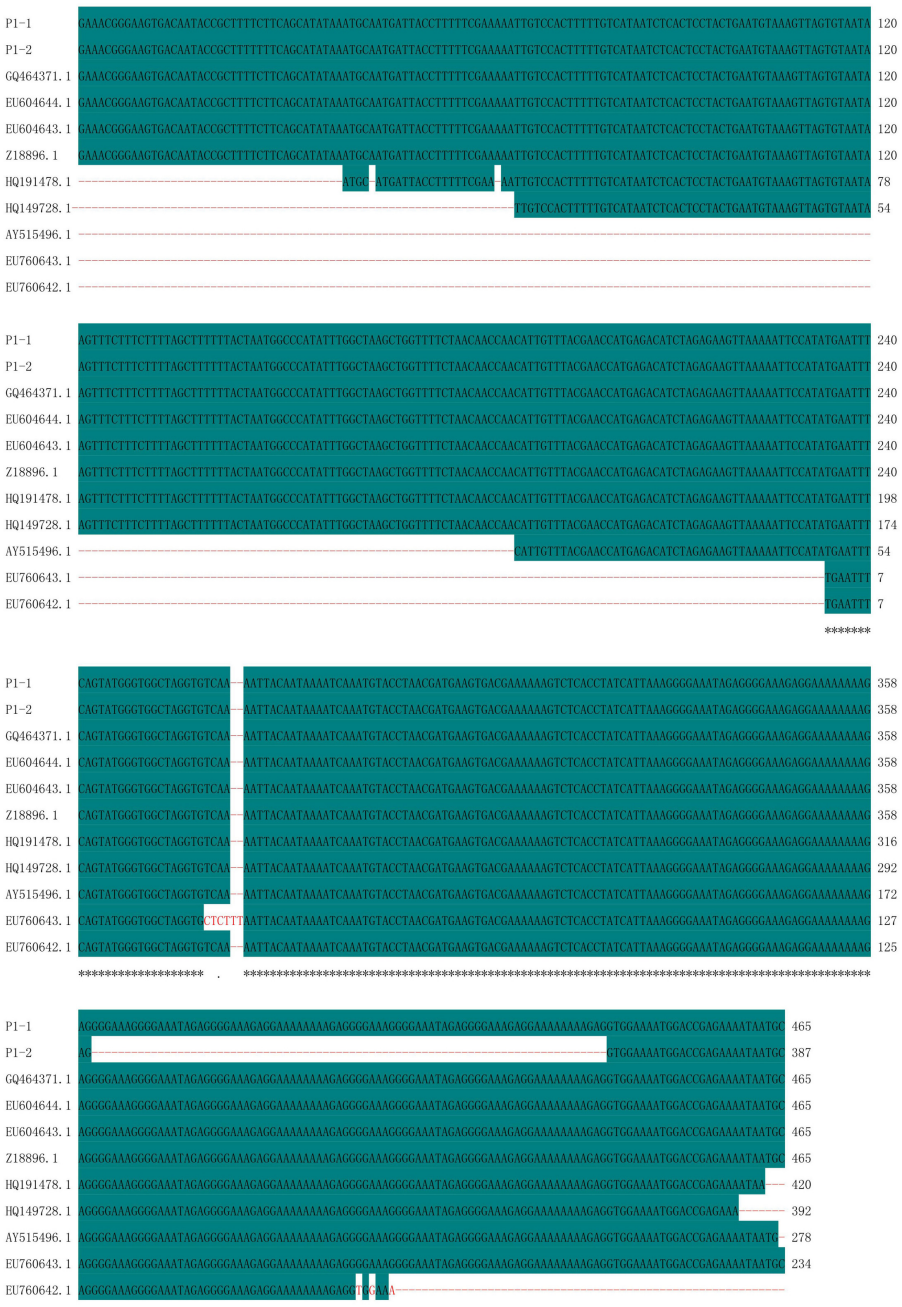
**Sequence alignment of amplicons produced by primer P1**. P1-1, 13CMS1–13CMS31 and 13CMS33–13CMS39; P1-2, 13CMS32. GQ464371.1 (*B. oleracea*), EU604644.1 (*B. oleracea* var. *italica*), EU604643.1 (*B. oleracea* var. *italica*), Z18896.1 (*R*. *sativus*), HQ191478.1 (*B. oleracea* var. *acephala*), HQ149728.1 (*B. oleracea* var. *italica*), AY515496.1 (*B. oleracea* var. *botrytis*), EU760643.1 (*B. oleracea* var. *capitata*), and EU760642.1 (*B. oleracea* var. *capitata*) were the related sequences in the GenBank. Asterisks means identical base.

Polymorphic amplicons obtained using seven polymorphic markers (P1, P8–P11, P15, and P16; Table 2) were sequenced and aligned. The amplicons generated by primer P1 for CMS32 were 387 bp, whereas those of the other CMS accessions were 465 bp. There was an 83.01% sequence similarity, with a single nucleotide polymorphism at position 28 (C/T). We also observed a 78-nucleotide deletion between positions 361 and 438 of 13CMS32 (Figure 3). The amplicons produced by primers P8–P11 also had a 78-nucleotide deletion at the same position as the *orf138*-related sequence in 13CMS32.

**Figure 4:**
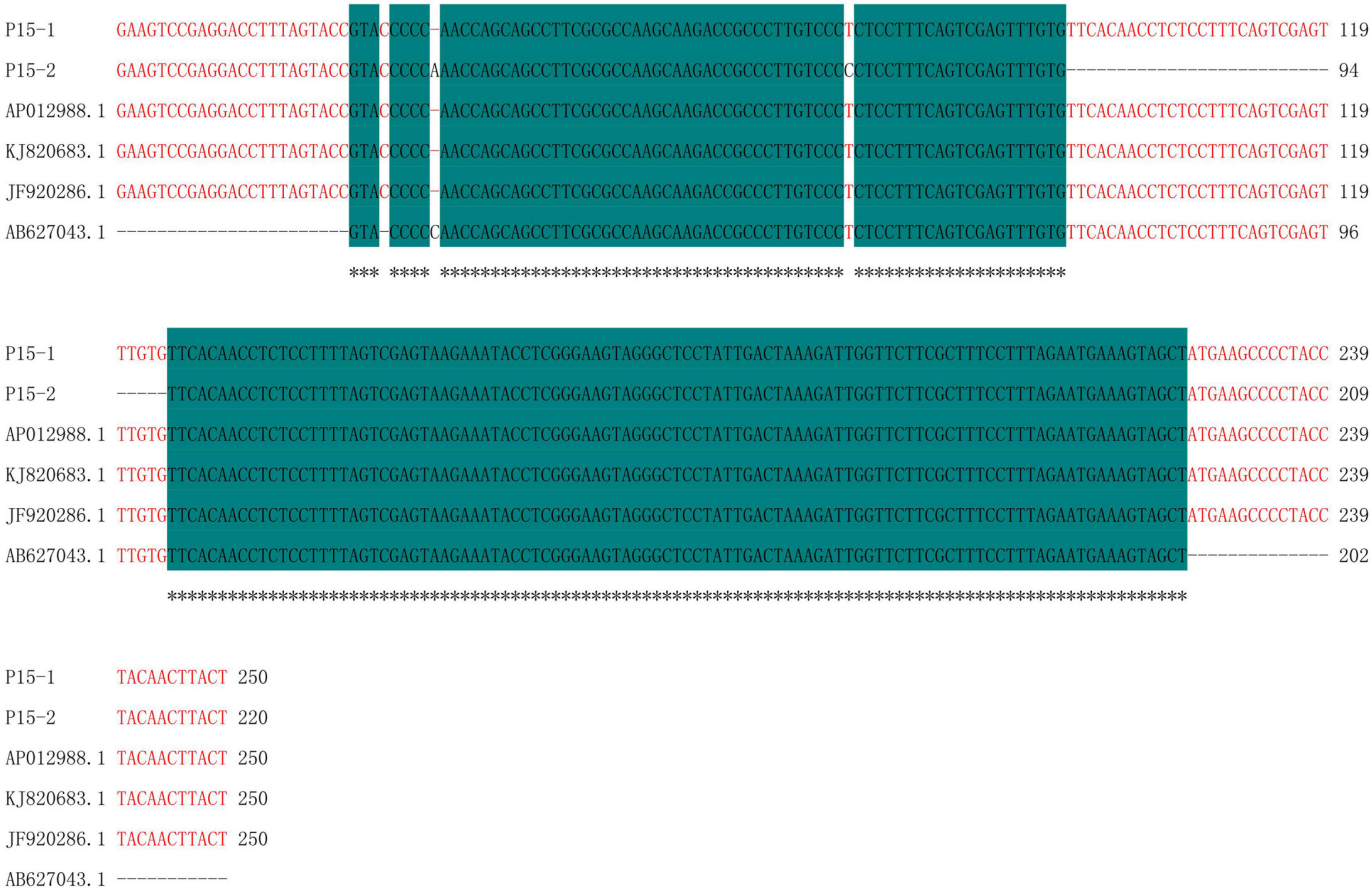
**Sequence alignment of amplicons produced by primer P15**. P15-1, 13CMS1–13CMS5, 13CMS7–13CMS22, 13CMS25–13CMS36, and 13CMS38; P15-2, 13CMS6, 13CMS23, 13CMS24, 13CMS37, and 13CMS39. AP012988.1 (*B. oleracea*), KJ820683.1 (*B. oleracea* var. *botrytis*), JF920286.1 (*B. oleracea*), and AB627043.1 (*B. oleracea*) were the related sequences in the GenBank. Asterisks means identical base.

The amplicons generated by primer P15 were 220 bp and identical for 13CMS6, 13CMS23, 13CMS24, 13CMS37, and 13CMS39. However, they were 250 bp for the other CMS accessions. We observed an 86.85% sequence similarity, with one nucleotide deleted at position 32 and a single nucleotide polymorphism at position 73 (T/C). Additionally, there was a 31-nucleotide deletion between positions 96 and 126 for 13CMS6, 13CMS23, 13CMS24, 13CMS37, and 13CMS39 (Figure 4).

**Figure 5:**
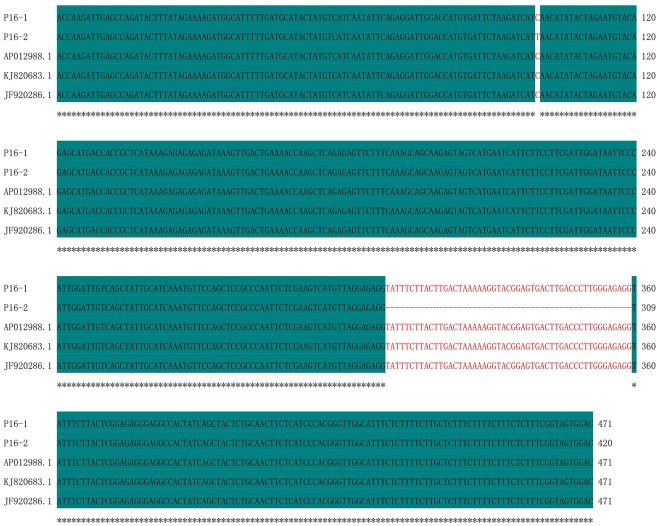
**Sequence alignment of amplicons produced by primer P16**. P16-1, 13CMS1–13CMS5, 13CMS7–13CMS22, 13CMS25–13CMS36, and 13CMS38; P16-2, 13CMS6, 13CMS23, 13CMS24, 13CMS37, and 13CMS39. AP012988.1 (*B. oleracea*), KJ820683.1 (*B. oleracea* var. *botrytis*) and JF920286.1 (*B. oleracea*) were the related sequences in the GenBank. Asterisks means identical base.

The amplicons produced by primer P16 were 420 bp and identical in 13CMS6, 13CMS23, 13CMS24, 13CMS37, and 13CMS39. They were 471 bp and identical in all other CMS accessions. The sequence similarity was 88.96%, with a single nucleotide polymorphism at position 100 (C/T). Additionally, there was a 51-nucleotide deletion between positions 309 and 359 for 13CMS6, 13CMS23, 13CMS24, 13CMS37, and 13CMS39 (Figure 5).

Similarity analysis of the sequences revealed that the amplicons of primer P1 have the highest similarity with *ogura* cytoplasmic male sterility-related protein gene in *B. oleracea, B. oleracea* var. *italic*, and *R*. *sativus* (Figure 3, Supplementary Table 2); the amplicons of primer P15 and P16 have high similarity with mitochondrial DNA in *B. oleracea* and *B. oleracea* var. *botrytis* (Figures 4, 5, Supplementary Tables 3, 4).

## Discussion

CMS, which is usually caused by mutations in the mitochondrial genome (Jing et al., 2015), is prevalent in higher plants and can be used to increase heterosis and improve genetic resources. Mitochondrial markers can be used to quickly and accurately differentiate between the various types of CMS sources (Zhao et al., 2010; Wang et al., 2012; Zhang et al., 2013). Protoplast fusion (Yarrow et al., 1990) and distant hybridization (Jing et al., 2015) have been the main approaches used to obtain CMS materials in broccoli. However, to the best of our knowledge, there are few reports describing the application of mitochondrial markers to identify the types of CMS resources. Furthermore, the genetic diversity of the sources of CMS in broccoli has not been determined. Previous reports were all based on the development of markers to differentiate between *ogu* CMS materials and fertile broccoli accessions (Zhu et al., 2006; Yao et al., 2009; Jing et al., 2015).

In this study, we used primers specific for *orf138, orf222, orf222-orf224, orf224, orf224-atp6, orf263*, and mitochondrial SSR markers to systematically determine the CMS origins of 39 broccoli accessions, which were obtained from various countries and regions. All 39 CMS accessions contained the CMS-related *orf138* fragment, and *orf222, orf222-orf224, orf224, orf224-atp6*, and *orf263* fragments could not be detected, suggesting these accessions expressed *ogu* CMS. This is consistent with previous reports that *ogu* CMS occurs in *Brassica* and *Raphanus* crops (Makaroff et al., 1989; Zhang et al., 2006; Chen et al., 2009). Our results are also consistent with those of previous studies on CMS sources in broccoli (Yao et al., 2009; Jing et al., 2015) and indicate that the CMS origin is single in broccoli and more types of CMS from related species should be introduced into broccoli in future breeding programs.

CMS1 consisted of *ogu* CMS R_3_, which originated from a somatic hybridization and was transferred from a cabbage CMS line. The type of CMS in CMS2–CMS39 could not be confirmed. We developed eight polymorphic cytoplasmic markers (P1, P8–P11, P13, P15, and P16; Table 2) in 39 broccoli CMS accessions (CMS1–CMS39) which could distinguish their cytoplasm types. Using the eight cytoplasmic markers, we divided these accessions into five groups (coefficient > 0.89) or three groups (coefficient > 0.745) according to cluster analysis data. When the coefficient was larger than 0.745, CMS32 formed its own group, CMS6, CMS23, CMS24, CMS37, and CMS39 belonged to one group, and the other 33 CMS accessions formed another group. The results were difference when the coefficient was larger than 0.89, CMS6, CMS26, and CMS32 each formed its own group, CMS23, CMS24, CMS37, and CMS39 belonged to one group, and the other 32 CMS accessions formed another group. These results suggesting lines CMS2–CMS5, CMS7–CMS22, CMS25, CMS27–CMS31, CMS33–CMS36, and CMS38 were included in the same group as CMS1 with high coefficient, and *ogu* CMS R_3_was likely the original CMS. These lines constituted 79.49% of the total CMS resources used in our study, demonstrating that *ogu* CMS R_3_ is prevalent in broccoli. It is likely that the original CMS source developed from a different protoplast fusion event and the resulting CMS was subsequently transferred to broccoli. When the coefficient is larger than 0.89, CMS6, CMS23, CMS24, CMS26, CMS37, CMS32, and CMS39 didn't belong to the same group with CMS1, implying they weren't *ogu* CMS R_3_type.

In this study, the amplicons of primer P16 were 420 bp and identical in lines CMS6, CMS23, CMS24, CMS37, and CMS39, they were 471 bp and identical in other 34 CMS accessions, the length of the polymorphic products were same as reported in cabbage, which can distinguish *ogu* CMS HY and *ogu* CMS R_1−2_ (420 bp) from *pol* CMS and *ogu* CMS R_3_ (471 bp; Wang et al., 2012). So our results suggest that the original of CMS6, CMS23, CMS24, CMS37, and CMS39 could come from *ogu* CMS HY or *ogu* CMS R_1−2_. Sequences analysis showed that the deleted sequences of these broccoli CMS accessions were not at the same location as in cabbage. Unfortunately, as far as we know, the genetic diversity of original sources of CMS in broccoli has not been documented and we have no references. In our study, the polymorphic cytoplasmic markers were less and maybe some accessions could not be distinguished, further experiments with more polymorphic markers and extensive research materials will deepen our understanding of the diversity in the cytoplasm types of CMS in broccoli.

When the coefficient (Figure 2) was about 0.90, CMS6, CMS23, CMS24, CMS37, and CMS39 formed one group. The flowers of the original broccoli lines of CMS6, CMS37, and CMS39 had carpelloid stamens and exhibited poor seed setting (Shu J. et al., 2015), which suggests that CMS23 and CMS24 also contain carpelloid stamens. Therefore, the CMS source for CMS6, CMS23, CMS24, CMS37, and CMS39 should not be transferred into broccoli lines. Lines CMS26 and CMS32 each formed its own group, indicating their *ogu* CMS was different from those of the other 37 CMS accessions. Our findings suggest that some of the original sources of *ogu* CMS were better than others. Alternatively, existing fragments were lost during CMS transfer, resulting in differences among the observed *ogu* CMS.

In conclusion, we have identified eight useful mitochondrial molecular markers to detect *ogu* CMS in different broccoli CMS accessions. The 39 CMS accessions could be divided into five groups (coefficient > 0.89) or three groups (coefficient > 0.745), *ogu* CMS R_3_ is prevalent in broccoli, *ogu* CMS HY or *ogu* CMS R_1−2_ is likely exist in broccoli. Our findings may help to improve the efficiency of CMS selection during broccoli breeding and also form a solid basis for further studies into the molecular mechanisms of *ogu* CMS in broccoli.

## Author contributions

YL and JS conceived and designed the study. JS and LZ performed the experiments. JS, ZL, LZ, and HL analyzed the data. JS prepared the manuscript, YL improved the manuscript. YL, ZF, LY, MZ, and YZ contributed reagents and materials. YL provided guidance on the whole study. All authors read and approved the final manuscript.

### Conflict of interest statement

The authors declare that the research was conducted in the absence of any commercial or financial relationships that could be construed as a potential conflict of interest.
