# Supplementary material for: Detection of the Diversity of Cytoplasmic Male Sterility Sources in Broccoli (Brassica Oleracea var. Italica) Using Mitochondrial Markers
Source: Front Plant Sci. 2016 Jun 24;7:927. doi: 10.3389/fpls.2016.00927 (PMC4919338; doi:10.3389/fpls.2016.00927)
Supplement: Supplementary file 1 [file DataSheet1.pdf]

Supplementary Material

Detection of the cytoplasmic male sterility sources in broccoli (*Brassica oleracea* var. *italica*) using mitochondrial markers

Jinshuai Shu, Yumei Liu[*], Zhansheng Li, Lili Zhang, Zhiyuan Fang, Limei Yang, Mu Zhuang, Yangyong Zhang, Honghao Lv

*** Correspondence:** Yumei Liu: liuyumei@caas.cn

Supplementary Tables

Supplementary Table 1 Broccoli CMS accessions and origins used for validation of the polymorphic markers in this study

Code	Line name	Type	Backcross generations	Origin of cytoplasmic male sterile sources
14CMS1	OguraCMS R_3-B59 [a]	Cytoplasmic male sterile line	BC_{17}	Variety introduction (Asgrow seed Co., US)
14CMS2	CMS04-B59	Cytoplasmic male sterile line	BC_{10}	Variety introduction (Shanghai Horticultural Research Institute, China)
14CMS3	CMS07-B59	Cytoplasmic male sterile line	BC_{10}	Variety introduction (Beijing Vegetable Research Center, China)
14CMS4	CMS12-B59	Cytoplasmic male sterile line	BC_8	Variety introduction (Shanghai Horticultural Research Institute, China)
14CMS5	CMS13-B59	Cytoplasmic male sterile line	BC_9	Variety introduction (Beijing Vegetable Research Center, China)
14CMS6	CMS132-B59	Cytoplasmic male sterile line	BC_9	Variety introduction (Japan)
14CMS7	CMS724-B59	Cytoplasmic male sterile line	BC_8	Variety introduction (Taiwan, China)
14CMS8	CMS727-B59	Cytoplasmic male sterile line	BC_9	Variety introduction (Seminis Seeds Co., Ltd., USA)
14CMS9	CMS736-B59	Cytoplasmic male sterile line	BC_8	Variety introduction (Japan)
14CMS10	CMS738-B59	Cytoplasmic male sterile line	BC_8	Variety introduction (Japan)
14CMS11	CMSGD-B59	Cytoplasmic male sterile line	BC_7	Variety introduction (Guangdong, China)
14CMS12	CMS1190-B59	Cytoplasmic male sterile line	BC_5	Variety introduction (Beijing Honor Seeds Co., Ltd., China)
14CMS13	CMS1162-B59	Cytoplasmic male sterile line	BC_5	Variety introduction (Kunming Kunhua Seed Co., Ltd., China)
14CMS14	CMS1166-B59	Cytoplasmic male sterile line	BC_5	Variety introduction (Kunming Kunhua Seed Co., Ltd., China)
14CMS15	CMS1169-B59	Cytoplasmic male sterile line	BC_5	Variety introduction (Kunming Kunhua Seed Co., Ltd., China)
14CMS16	CMS1176-B59	Cytoplasmic male sterile line	BC_5	Variety introduction (Kunming Kunhua Seed Co., Ltd., China)
14CMS17	CMS1177-B59	Cytoplasmic male sterile line	BC_5	Variety introduction (Kunming Kunhua Seed Co., Ltd., China)
14CMS18	CMS1183-B59	Cytoplasmic male sterile line	BC_5	Variety introduction (Beijing, China)
14CMS19	CMSYB6-B59	Cytoplasmic male sterile line	BC_3	Variety introduction (Japan)
14CMS20	CMSLvXinErHao-B59	Hybrid	-	Variety introduction (Taiwan Suntech Seed Co., Ltd., China)
14CMS21	CMSYouSheng-B59	Hybrid	-	Variety introduction (Wong Ching Ho Co., Ltd., Hong Kong, China)

14CMS22	CMSYuXi-B59	Hybrid	-	Variety introduction (Kunming Kunhua Seed Co., Ltd., China)
14CMS23	CMSWeiJingLv-B59	Hybrid	-	Variety introduction (Tianhe agricultural companies, Hong Kong, China)
14CMS24	CMSWangLv-B59	Hybrid	-	Variety introduction (Japan)
14CMS25	CMSB2944-B59	Hybrid	-	Variety introduction (Wei Qin Enterprises Ltd., Hong Kong, China)
14CMS26	CMSMarathon-B59	Hybrid	-	Variety introduction (Japan)
14CMS27	CMSB2946-B59	Hybrid	-	Variety introduction (Beijing Honor Seeds Co., Ltd., Beijing, China)
14CMS28	CMSB2947-B59	Hybrid	-	Variety introduction (Beijing Honor Seeds Co., Ltd., Beijing, China)
14CMS29	CMSLD66-B59	Hybrid	-	Variety introduction (Korea)
14CMS30	CMSB2949-B59	Hybrid	-	Variety introduction (Tianjin Kernel Vegetable Research Institute, China)
14CMS31	CMSB2950-B59	Hybrid	-	Variety introduction (Tianjin Kernel Vegetable Research Institute, China)
14CMS32	CMSXiLanHuaErHao-B59	Hybrid	-	Variety introduction (Seminis Seeds Co., Ltd., USA)
14CMS33	CMSB2952-B59	Hybrid	-	Variety introduction (Wong Ching Ho Co., Ltd., Hong Kong, China)
14CMS34	CMS YouXiu-B59	Hybrid	-	Variety introduction (Japan)
14CMS35	CMSB2069-B59	Hybrid	-	Variety introduction (Sakata Seed Corporation, Japan)
14CMS36	CMSB2071-B59	Hybrid	-	Variety introduction (Seminis Seeds Beijing Co., Ltd., Beijing, China)
14CMS37	CMSB2072-B59	Hybrid	-	Variety introduction (Taiwan Ho-Huan Agricultural Product Co., Ltd., China)
14CMS38	CMSB2074-B59	Hybrid	-	Variety introduction (Syngenta China Company, Beijing, China)
14CMS39	CMSB2075-B59	Hybrid	-	Variety introduction (Seminis Seeds Beijing Co., Ltd., Beijing, China)
14ML	B59	Inbred	-	Institute of Vegetables and Flowers, Chinese Academy of Agricultural Sciences (Beijing, China)
15CMS1	OguraCMS R_3-B59	Cytoplasmic male sterile line	BC_{18}	Variety introduction (Asgrow seed Co., US)
15CMS2	CMS04-B59	Cytoplasmic male sterile line	BC_{11}	Variety introduction (Shanghai Horticultural Research Institute, China)
15CMS3	CMS07-B59	Cytoplasmic male sterile line	BC_{11}	Variety introduction (Beijing Vegetable Research Center, China)
15CMS4	CMS12-B59	Cytoplasmic male sterile line	BC_9	Variety introduction (Shanghai Horticultural Research Institute, China)
15CMS5	CMS13-B59	Cytoplasmic male sterile line	BC_{10}	Variety introduction (Beijing Vegetable Research Center, China)

15CMS6	CMS132-B59	Cytoplasmic male sterile line	BC_{10}	Variety introduction (Japan)
15CMS7	CMS724-B59	Cytoplasmic male sterile line	BC_9	Variety introduction (Taiwan, China)
15CMS8	CMS727-B59	Cytoplasmic male sterile line	BC_{10}	Variety introduction (Seminis Seeds Co., Ltd., USA)
15CMS9	CMS736-B59	Cytoplasmic male sterile line	BC_9	Variety introduction (Japan)
15CMS10	CMS738-B59	Cytoplasmic male sterile line	BC_9	Variety introduction (Japan)
15CMS11	CMSGD-B59	Cytoplasmic male sterile line	BC_8	Variety introduction (Guangdong, China)
15CMS12	CMS1190-B59	Cytoplasmic male sterile line	BC_6	Variety introduction (Beijing Honor Seeds Co., Ltd., China)
15CMS13	CMS1162-B59	Cytoplasmic male sterile line	BC_6	Variety introduction (Kunming Kunhua Seed Co., Ltd., China)
15CMS14	CMS1166-B59	Cytoplasmic male sterile line	BC_6	Variety introduction (Kunming Kunhua Seed Co., Ltd., China)
15CMS15	CMS1169-B59	Cytoplasmic male sterile line	BC_6	Variety introduction (Kunming Kunhua Seed Co., Ltd., China)
15CMS16	CMS1176-B59	Cytoplasmic male sterile line	BC_6	Variety introduction (Kunming Kunhua Seed Co., Ltd., China)
15CMS17	CMS1177-B59	Cytoplasmic male sterile line	BC_6	Variety introduction (Kunming Kunhua Seed Co., Ltd., China)
15CMS18	CMS1183-B59	Cytoplasmic male sterile line	BC_6	Variety introduction (Beijing, China)
15CMS19	CMSYB6-B59	Cytoplasmic male sterile line	BC_4	Variety introduction (Japan)
15CMS20	CMSLvXinErHao-B59	Cytoplasmic	BC_1	Variety introduction (Taiwan Suntech Seed Co., Ltd., China)
15CMS21	CMSYouSheng-B59	Cytoplasmic	BC_1	Variety introduction (Wong Ching Ho Co., Ltd., Hong Kong, China)
15CMS22	CMSYuXi-B59	Cytoplasmic	BC_1	Variety introduction (Kunming Kunhua Seed Co., Ltd., China)
15CMS23	CMSWeiJingLv-B59	Cytoplasmic	BC_1	Variety introduction (Tianhe agricultural companies, Hong Kong, China)
15CMS24	CMSWangLv-B59	Cytoplasmic	BC_1	Variety introduction (Japan)
15CMS25	CMSB2944-B59	Cytoplasmic	BC_1	Variety introduction (Wei Qin Enterprises Ltd., Hong Kong, China)
15CMS26	CMSMarathon-B59	Cytoplasmic	BC_1	Variety introduction (Japan)
15CMS27	CMSB2946-B59	Cytoplasmic	BC_1	Variety introduction (Beijing Honor Seeds Co., Ltd., Beijing, China)
15CMS28	CMSB2947-B59	Cytoplasmic	BC_1	Variety introduction (Beijing Honor Seeds Co., Ltd., Beijing, China)
15CMS29	CMSLD66-B59	Cytoplasmic	BC_1	Variety introduction (Korea)

15CMS30	CMSB2949-B59	Cytoplasmic	BC_1	Variety introduction (Tianjin Kernel Vegetable Research Institute, China)
15CMS31	CMSB2950-B59	Cytoplasmic	BC_1	Variety introduction (Tianjin Kernel Vegetable Research Institute, China)
15CMS32	CMSXiLanHuaErHao-B59	Cytoplasmic	BC_1	Variety introduction (Seminis Seeds Co., Ltd., USA)
15CMS33	CMSB2952-B59	Cytoplasmic	BC_1	Variety introduction (Wong Ching Ho Co., Ltd., Hong Kong, China)
15CMS34	CMS YouXiu-B59	Cytoplasmic	BC_1	Variety introduction (Japan)
15CMS35	CMSB2069-B59	Cytoplasmic	BC_1	Variety introduction (Sakata Seed Corporation, Japan)
15CMS36	CMSB2071-B59	Cytoplasmic	BC_1	Variety introduction (Seminis Seeds Beijing Co., Ltd., Beijing, China)
15CMS37	CMSB2072-B59	Cytoplasmic	BC_1	Variety introduction (Taiwan Ho-Huan Agricultural Product Co., Ltd., China)
15CMS38	CMSB2074-B59	Cytoplasmic	BC_1	Variety introduction (Syngenta China Company, Beijing, China)
15CMS39	CMSB2075-B59	Cytoplasmic	BC_1	Variety introduction (Seminis Seeds Beijing Co., Ltd., Beijing, China)
15ML	B59	Cytoplasmic	-	Institute of Vegetables and Flowers, Chinese Academy of Agricultural Sciences (Beijing, China)

[a]Number refers to the genotype of the backcross parents; - : no backcross generations.

Supplementary Table 2 Sequence identity between amplified products of primer P1 in broccoli CMS accessions and the corresponding sequences of *B. oleracea*, *B. oleracea* var. *italica*, *B. oleracea* var. *botrytis*, *R. sativus*, *B. oleracea* var. *acephala*, and *B. oleracea* var. *capitata* cytoplasmic genomes

Accession	Organism	Description Protein name	Sequence identity (P1-1)	Sequence identity (P1-2)
GQ464371.1	*B. oleracea*	Mitochondrial *ogura* cytoplasmic male sterility-related protein gene, complete cds; nuclear gene for mitochondrial product	100%	83.011%
EU604644.1	*B. oleracea* var. *italica*	Cultivar NAU-Y04A tRNA-Met (trnfM) gene, complete sequence; and *ogura* cytoplasmic male sterility-related protein gene, complete cds; mitochondrial	100%	83.011%
EU604643.1	*B. oleracea* var. *italica*	Cultivar NAU-Lb03A tRNA-Met (trnfM) gene, complete sequence; and *ogura* cytoplasmic male sterility-related protein gene, complete cds; mitochondrial	100%	83.011%
Z18896.1	*R. sativus*	Mitochondrion *orf138*, *orfB* and *trnfM* genes	100%	83.011%
HQ191478.1	*B. oleracea* var. *acephala*	Strain A1_pf cytoplasmic male sterility-related protein gene, complete cds	90.323%	73.118%
HQ149728.1	*B. oleracea* var. *italica*	Mitochondrial ogura cytoplasmic male sterility-related protein gene, partial cds; nuclear gene for mitochondrial product	84.301%	67.097%
AY515496.1	*B. oleracea* var. *botrytis*	Clone NH86 (26-1) NH86P male sterility marker; mitochondrial	59.785%	42.796%
EU760643.1	*B. oleracea* var. *capitata*	Cytoplasmic male sterility protein 60 gene, complete cds; mitochondrial	48.822%	32.12%
EU760642.1	*B. oleracea* var. *capitata*	Cytoplasmic male sterility protein 67 gene, partial cds; mitochondrial	36.559%	35.222%

P1-1: 13CMS1–13CMS31 and 13CMS33–13CMS39; P1-2: 13CMS32.

Supplementary Table 3 Sequence identity between amplified products of primer P15 in broccoli CMS accessions and the corresponding sequences of *B. oleracea* and *B. oleracea* var. *botrytis* cytoplasmic genomes

Accession	Organism	Description Protein name	Sequence identity (P15-1)	Sequence identity (P15-2)
AP012988.1	*B. oleracea*	Mitochondrial DNA, complete sequence, cultivar: Fujiwase	100%	86.853%
KJ820683.1	*B. oleracea* var. *botrytis*	Mitochondrion, complete genome	100%	86.853%
JF920286.1	*B. oleracea*	Mitochondrial DNA, complete genome	100%	86.853%
AB627043.1	*B. oleracea*	Mitochondrial DNA, minisatellite: BnTR4	80.8%	67.331%

P15-1: 13CMS1–13CMS5, 13CMS7–13CMS22, 13CMS25–13CMS36, and 13CMS38; P15-2: 13CMS6, 13CMS23, 13CMS24, 13CMS37, and 13CMS39.

Supplementary Table 4 Sequence identity between amplified products of primer P15 in broccoli CMS accessions and the corresponding sequences of *B. oleracea* and *B. oleracea* var. *botrytis* cytoplasmic genomes

Accession	Organism	Description Protein name	Sequence identity (P16-1)	Sequence identity (P16-2)
AP012988.1	*B. oleracea*	Mitochondrial DNA, complete sequence, cultivar: Fujiwase	100%	88.96%
KJ820683.1	*B. oleracea* var. *botrytis*	Mitochondrion, complete genome	100%	88.96%
JF920286.1	*B. oleracea*	Mitochondrial DNA, complete genome	100%	88.96%

P16-1: 13CMS1–13CMS5, 13CMS7–13CMS22, 13CMS25–13CMS36, and 13CMS38; P16-2: 13CMS6, 13CMS23, 13CMS24, 13CMS37, and 13CMS39.

Sequence1 Amplicons of primer P1 in 13CMS1–13CMS31 and 13CMS33–13CMS39
GAAACGGGAAGTGACAATACCGCTTTTCTTCAGCATATAAATGCAATGATTACCTTTTTCGAAAAATT
GTCCACTTTTTGTCATAATCTCACTCCTACTGAATGTAAAGTTAGTGTAATAAGTTTCTTTCTTTTAGCT
TTTTTACTAATGGCCCATATTTGGCTAAGCTGGTTTTCTAACAACCAACATTGTTTACGAACCATGAGA
CATCTAGAGAAGTTAAAAATTCCATATGAATTTCAGTATGGGTGGCTAGGTGTCAAAATTACAATAAA
ATCAAATGTACCTAACGATGAAGTGACGAAAAAAGTCTCACCTATCATTAAAGGGGAAATAGAGGGG
AAAGAGGAAAAAAAAGAGGGGAAAGGGGAAATAGAGGGGAAAGAGGAAAAAAAAGAGGGGAAAG
GGGAAATAGAGGGGAAAGAGGAAAAAAAAGAGGTGGAAAATGGACCGAGAAAATAATGC

Sequence2 Amplicons of primer P1 in 13CMS32
GAAACGGGAAGTGACAATACCGCTTTTTTTCAGCATATAAATGCAATGATTACCTTTTTCGAAAAATT
GTCCACTTTTTGTCATAATCTCACTCCTACTGAATGTAAAGTTAGTGTAATAAGTTTCTTTCTTTTAGCT
TTTTTACTAATGGCCCATATTTGGCTAAGCTGGTTTTCTAACAACCAACATTGTTTACGAACCATGAGA
CATCTAGAGAAGTTAAAAATTCCATATGAATTTCAGTATGGGTGGCTAGGTGTCAAAATTACAATAAA
ATCAAATGTACCTAACGATGAAGTGACGAAAAAAGTCTCACCTATCATTAAAGGGGAAATAGAGGGG
AAAGAGGAAAAAAAAGAGGTGGAAAATGGACCGAGAAAATAATGC

Sequence3 Amplicons of primer P15 in 13CMS1–13CMS5, 13CMS7–13CMS22, 13CMS25–13CMS36, and 13CMS38
GAAGTCCGAGGACCTTTAGTACCGTACCCCCAACCAGCAGCCTTCGCGCCAAGCAAGACCGCCCTTGT
CCCTCTCCTTTCAGTCGAGTTTGTGTTCACAACCTCTCCTTTCAGTCGAGTTTGTGTTCACAACCTCTCC
TTTTAGTCGAGTAAGAAATACCTCGGGAAGTAGGGCTCCTATTGACTAAAGATTGGTTCTTCGCTTTCC
TTTAGAATGAAAGTAGCTATGAAGCCCCTACCTACAACTTACT

Sequence4 Amplicons of primer P15 in 13CMS6, 13CMS23, 13CMS24, 13CMS37, and 13CMS39
GAAGTCCGAGGACCTTTAGTACCGTACCCCCAAACCAGCAGCCTTCGCGCCAAGCAAGACCGCCCTT
GTCCCCCTCCTTTCAGTCGAGTTTGTGTTCACAACCTCTCCTTTTAGTCGAGTAAGAAATACCTCGGGA
AGTAGGGCTCCTATTGACTAAAGATTGGTTCTTCGCTTTCCTTTAGAATGAAAGTAGCTATGAAGCCC
CTACCTACAACTTACT

Sequence5 Amplicons of primer P16 in 13CMS1–13CMS5, 13CMS7–13CMS22, 13CMS25–13CMS36, and 13CMS38
ACCAAGATTGAGCCAGATACTTTATAGAAAAGATGGCATTTTTGA**TGCATACTATGTCATCAA**TATT
CAGAGGATTGGACCATGTGATTCTAAGATCAT**C**AACATATACTAGAATGTACAGAGCATGACCACCG
CTCATAAAGAGAGAGAGATAAAGTTGACTGAAAACCAAGCTCAGAGAGTTCTTTCAAAGCAGCAAGA
GTAGTCATGAATCATTCTTCCTTCGATTGGATAATTCCCATTGGATTGTCAGCTATTGCATCAAATGTT
CCAGCTCCGCCCAATTCTCGAAGTCATGTTAGGAGAGGTATTTCTTACTTGACTAAAAAGGTACGGAG
TGACTTGACCCTTGGGAGAGGTATTTCTTACTCGGAGAGGGAGGCCACTATCAGCTACTCTGCAACTT
CTCATCCCACGGGTTGGCATTTCTCTTTTCTTGCTCTTTCTTTTCTT**TCTCTTT**CGGTAGTGGAC

Sequence6 Amplicons of primer P16 in 13CMS6, 13CMS23, 13CMS24, 13CMS37, and 13CMS39
ACCAAGATTGAGCCAGATACTTTATAGAAAAGATGGCATTTTTGA**TGCATACTATGTCATCAA**TATT
CAGAGGATTGGACCATGTGATTCTAAGATCAT**T**AACATATACTAGAATGTACAGAGCATGACCACCG
CTCATAAAGAGAGAGAGATAAAGTTGACTGAAAACCAAGCTCAGAGAGTTCTTTCAAAGCAGCAAGA
GTAGTCATGAATCATTCTTCCTTCGATTGGATAATTCCCATTGGATTGTCAGCTATTGCATCAAATGTT
CCAGCTCCGCCCAATTCTCGAAGTCATGTTAGGAGAGGTATTTCTTACTCGGAGAGGGAGGCCACTAT
CAGCTACTCTGCAACTTCTCATCCCACGGGTTGGCATTTCTCTTTTCTTGCTCTTTCTTTTCTTTCTCTTT
CGGTAGTGGAC
